# Using participatory design to develop (public) health decision support systems through GIS

**DOI:** 10.1186/1476-072X-6-53

**Published:** 2007-11-27

**Authors:** S Michelle Dredger, Anita Kothari, Jason Morrison, Michael Sawada, Eric J Crighton, Ian D Graham

**Affiliations:** 1 Department of Community Health Sciences, University of Manitoba, S113-750 Bannatyne Ave, Winnipeg, Canada; 2 Faculty of Health Sciences, Bachelor of Health Sciences, University of Western Ontario. Arthur & Sonia Labatt Health Sciences Building, Room 222, London, Canada; 3 Department of Biosystems Engineering, University of Manitoba, E2-376 Engineering Building, University of Manitoba, Winnipeg, Canada; 4 Laboratory for Applied Geomatics and GIS Science (LAGGISS), Department of Geography, University of Ottawa, Ottawa, Canada; 5 Department of Geography, University of Ottawa, Ottawa, Canada; 6 School of Nursing and Dept of Epidemiology & Community Medicine, University of Ottawa, 451 Smyth Road, Ottawa, Canada; 7 Affiliate Scientist, Clinical Epidemiology Program, Ottawa Health Research Institute, 725 Parkdale Ave., Ottawa, Canada; 8 VP Knowledge Translation, Canadian Institutes of Health Research, 160 Elgin Street, Ottawa, Canada

## Abstract

**Background:**

Organizations that collect substantial data for decision-making purposes are often characterized as being 'data rich' but 'information poor'. Maps and mapping tools can be very useful for research transfer in converting locally collected data into information. Challenges involved in incorporating GIS applications into the decision-making process within the non-profit (public) health sector include a lack of financial resources for software acquisition and training for non-specialists to use such tools. This on-going project has two primary phases. This paper critically reflects on Phase 1: the participatory design (PD) process of developing a collaborative web-based GIS tool.

**Methods:**

A case study design is being used whereby the case is defined as the data analyst and manager dyad (a two person team) in selected Ontario Early Year Centres (OEYCs). Multiple cases are used to support the reliability of findings. With nine producer/user pair participants, the goal in Phase 1 was to identify barriers to map production, and through the participatory design process, develop a web-based GIS tool suited for data analysts and their managers. This study has been guided by the Ottawa Model of Research Use (OMRU) conceptual framework.

**Results:**

Due to wide variations in OEYC structures, only some data analysts used mapping software and there was no consistency or standardization in the software being used. Consequently, very little sharing of maps and data occurred among data analysts. Using PD, this project developed a web-based mapping tool (EYEMAP) that was easy to use, protected proprietary data, and permit limited and controlled sharing between participants. By providing data analysts with training on its use, the project also ensured that data analysts would not break cartographic conventions (e.g. using a chloropleth map for count data). Interoperability was built into the web-based solution; that is, EYEMAP can read many different standard mapping file formats (e.g. ESRI, MapInfo, CSV).

**Discussion:**

Based on the evaluation of Phase 1, the PD process has served both as a facilitator and a barrier. In terms of successes, the PD process identified two key components that are important to users: increased data/map sharing functionality and interoperability. Some of the challenges affected developers and users; both individually and as a collective. From a development perspective, this project experienced difficulties in obtaining personnel skilled in web application development and GIS. For users, some data sharing barriers are beyond what a technological tool can address (e.g. third party data). Lastly, the PD process occurs in real time; both a strength and a limitation. Programmatic changes at the provincial level and staff turnover at the organizational level made it difficult to maintain buy-in as participants changed over time. The impacts of these successes and challenges will be evaluated more concretely at the end of Phase 2.

**Conclusion:**

PD approaches, by their very nature, encourage buy-in to the development process, better addresses user-needs, and creates a sense of user-investment and ownership.

## Introduction

Health services agencies tend to be data-rich, but information-poor [1]. While health services research produces findings that should improve the quality of care, services and policies, consideration of the relevant research is not always evident. A consistent finding in health services research is that the transfer of research findings into practice is somewhat random and disorganized [2,3]. Moreover, empirical studies in the social sciences in general demonstrate that research rarely affects public policy [4-7].

Maps represent an innovative solution to assist the uptake of research evidence in decision-making. The usefulness of maps and mapping software as visual data dissemination tools has been recognized both in health and in other domains (e.g. transportation, crime analysis) because larger amounts of data can be presented unambiguously on a single page (e.g. [8]). This notion of information content is summarized succinctly by a common Geographic Information Systems (GIS) idiom; if a picture is worth a 1000 words then a map is worth a 1000 pictures. Maps and web-based mapping software have been used within western/developed country health research contexts in several capacities: to monitor disease surveillance trends (e.g. [9]); to increase ambulance response times by determining most efficient travel patterns (e.g. [10]); and to assess the distribution of health services facilities with respect to the population served [11,12]. These examples illustrate the potential for maps and mapping to rapidly and effectively disseminate health data (e.g. [13,14]).

The use of GIS and maps in health services planning and decision-making is limited by a number of significant barriers. These include a lack of financial resources for software acquisition and training, and the complexity of many of the market-based GIS software packages [15]. The effect of such barriers is particularly evident within the non-profit (public) health sector as in the example of Ontario Early Year's Centres (OEYCs), who's mandate is to assist in improving child developmental health (see [16]) through the provision of programs and services to parents and caregivers. This paper describes an ongoing project to evaluate the extent that web-based mapping software and maps – as tools for research transfer – can be used to support evidence-based decision-making for program planning and policies in OEYCs, and perhaps within the health services sector more generally. While OEYCs could benefit greatly from the use of maps in the decision-making process, if GIS tools remain in the hands of the few specialists, then the true benefits of mapping will remain glaringly absent.

The focus of this paper is on Phase 1 of the project, the collaborative and participatory design process used to develop a web-based GIS tool, called EYEMAP, to meet the established requirements of OEYC. Defined, participatory design (PD) includes a set of theories and practices that integrate end-users throughout the process culminating in computer software or hardware products and/or computer-based activities [17-20]. A value-added feature of this project is its focus on two levels of participation. Phase 1 includes the participatory design of the software, EYEMAP. Phase 2 looks at the integration of a series of activities designed to encourage the uptake and use of maps in decision-making processes; an activity that is also referred to as 'participatory GIS' [21]. While the focus for this paper is on the first level of participation – through the participatory design of the software – this critical reflection on the process signals possible issues and concerns to be addressed within Phase 2 of this research.

## Background

### Research Transfer and GIS

Conceptual models of research transfer have evolved to reflect the process of research uptake and utilization, such as diffusion models [22,23] and dissemination models [24]. These early models emphasize unidirectional information flow (from scientist to user) rather than interactive information exchange [25]. Later models of research transfer have, instead, supported the idea of "interaction" between producers/scientists and users of research for increased uptake of research findings [26,27]. Huberman [27,28] coined the terms *linkages *through *sustained interactivity*, leading to increased sensitivity about the user's domain. In this way, scientists can relate the research findings to organizational circumstances, or fine-tune implementation of research findings such that they are most likely to be incorporated into users' daily activities. In short, the interactive information exchange will produce the most relevant data for users, thereby enabling users to engage more fully with the findings [28].

Since the mid 1990s, GIS, the largest component of the field of Geomatics, has enjoyed rapid acceptance within the health planning, policy and analysis fields [29]. In these fields GIS has been collectively termed GIS-H (GIS-Health systems) [30]. Currently, within the Canadian and American health sectors, GIS and GIS-H (web-based) applications are used for rapid and effective analysis and dissemination of community health data [14,31] in multiple ways: facilities management and patient services (e.g. [32,33]); disease surveillance [9]; health care planning [33]; as well as data sharing and dissemination [31,32]. Moreover, with web-based GIS, the end-user does not require the technical expertise necessary to assemble and maintain complex spatial databases, which can be a substantial barrier to the adoption of GIS [14]. In web-based solutions, all thematic data (census boundaries, street-networks, socioeconomic data etc.) can be maintained on the server-side, freeing the client from data formatting, processing and maintenance issues. In general, for health services planning, web-based maps can present information in a more policy relevant and more easily interpreted format than non-spatial formats [8]. Boulos [14] notes that GIS in general offers a rich set of tools and methods that when properly designed and used can contribute positively to informed decision-making and planning of community health. Lack of effective training and therefore understanding on the side of personnel and analysts who wish to utilize GIS as a research transfer tool has been termed "spatial illiteracy" [30] in the GIS-H field. Spatial illiteracy describes a certain lack of knowledge of spatial data, maps and GIS among health professionals. This barrier means that spatial data users do not have a sufficient understanding of the most appropriate methodological approaches to address spatial questions or analyze mapped patterns [30]. Along with training, spatial illiteracy can be overcome by intelligent software design. For example, intelligent software can contain pre-packaged mapping methodologies for common health planning/analysis questions (e.g. software wizards) and would guide inexperienced users through common analytical processes [30].

### Participatory Design

Participatory design (PD) and collaborative prototyping are techniques that can assist in the development of computer software (also referred to as 'computer-artifacts') [20] that meets the needs of end-users. Designed to have computer software programs fit and transform the types of activities performed within an organization, the emphasis of PD is to elicit local knowledge and skills. In essence, it permits a collective process of learning and design [34]. Although PD emphasizes mutuality and reciprocity, traditional PD methods tend to be uni-directional: developers collect data and analyze the requirements *from *users; developers deliver a system *to *users [20]. Muller [20] extends these ideas within the field of human computer interaction (HCI) to examine a 'third space'; a hybrid realm that overlaps the work domains of a software professional and the end-users.

Exploring this 'third space' through PD can facilitate capacity building in both users and developers. Capacity building is defined here as encompassing the multiple dimensions of will, knowledge, skills, partnerships, resources, infrastructure, and leadership [35-37] needed to enhance an organization's ability to plan, implement, evaluate and sustain health promotion efforts [37,38]. Applied to this research project, developers are able to better understand and in turn integrate the types of tasks users need to perform within their organization, and users, through their interactions with developers, gain an understanding of the development process and the computational limits to their requests. The results of this process include not only a better, more tailored end product, but also a greater likelihood of user buy-in.

This 'third space' extension is embodied in earlier works in the PD and HCI traditions that examine the two paradigms between 'product-oriented' (where the computer artifact is seen as an end in itself) and 'process-oriented' (where the focus is on human work processes and the computer artifact serves as a means to reach the human goal) (see [39]). Embodied in these paradigms is the need for mutual learning among users and developers [40]. Moreover, this extension is relevant to this research project through the project's dual focus: from Phase 1, in developing a web-based software that meets the needs of data analysts (the product-oriented paradigm), to Phase 2, in providing the necessary support and training to encourage the uptake and use of maps as a decision-support system through a collaborative GIS (the process-oriented paradigm).

### Organizational Context for Study

**Figure 1:**
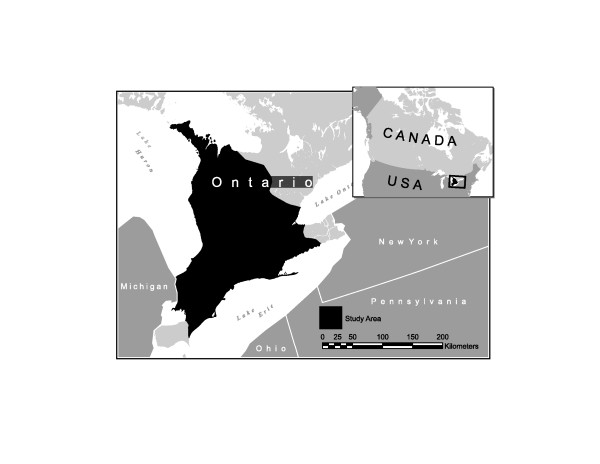


The organizational context for this project is the Ontario Early Years Centres (OEYCs) in Canada. Canada's federal system consists of ten provinces and three territories that are all signatories on the September 2000 Early Child Development Agreement designed to improve the health of children [41-43]. Ontario is one province that has added substantial provincial funding through the OEYCs to provide programs and services aimed at parents/caregivers with children under six to improve the developmental health of children (see [16]). Figure 1 shows a map of Canada and highlights the general location of our study participants in Ontario (for reasons of participant confidentiality specific regions are not identified).

The OEYCs serve as a focal point for local communities, facilitating the coordination of different groups and agencies to come together through four key areas: promoting healthy pregnancy, birth and infancy; improving parenting and family support; strengthening early childhood development, learning and care; and strengthening community support. Beginning in 2002, the OEYC program initially consisted of 15 communities. Today, OEYCs involve 103 communities, and are now under the organizational umbrella of the provinces. Recently, the province has implemented the Best Start early learning and care hubs in four demonstration sites. These hubs, located in accessible locations within communities, have been set up to provide families with a single, integrated, seamless point of access to services and supports. The services in a hub are based on local needs and community resources. OEYCs are now part of the Best Start initiative due to both their early year's programs and community planning capacity. Web-based mapping tools developed for OEYCs will support the type of community level planning needed for Best Start.

OEYCs have developed within a unique context that will have implications for future provincial roll-out of these Best Start integrated service hubs. Some OEYCs are part of their own administrative structure, while others are housed within municipal offices or public health units. These different organizational structures create a series of capacity-building challenges. Among these are the following: 1) differential resource support (i.e., OEYCs in municipalities may have access to GIS departments to do mapping for them); 2) variability in human resources and skills (i.e., data analysts have variable skill sets related to computer use, data collection, analysis and synthesis skills across sites); and/or 3) differential access to data (i.e., an OEYC in a municipality or a public health unit has potentially greater access to schools data, census data, etc compared to an OEYC in its own administrative unit that may have to purchase these data).

OEYCs are comprised of information/research producer (data analyst) and user (manager) pairs working in close proximity. OEYC data analysts have the responsibility to maintain and update a community service inventory (e.g., recreation sites, libraries, child development programmes). They also promote an Early Development Instrument in partnership with local school boards to assess a child's readiness to learn. The OEYCs and their analysts are to be a "valuable resource" to the communities they serve, and a "clearing house" for information on Early Years in their community [44]. Although OEYCs are strongly encouraged to produce maps, without a common platform, these producer/user dyads find it difficult to share information with their counterparts in other OEYCs for the purposes of public program planning and decision-making. Many OEYCs do not have ready access to some of the market-based GIS software packages, either due to expense or difficulty in using these packages. Consequently, many OEYCs lack the capacity to produce maps themselves, or they are required to rely on a department within their organization to produce maps for them. Either way, most OEYCs have not had the autonomy to use maps and mapping tools to their full potential. It is important to recall the variability that exists among OEYCs depending on their administrative unit that may exacerbate or minimize these challenges.

## Methods

In 2004, prior to the commencement of the project reported here, the research team administered a survey related to research transfer and mapping use in selected OEYCs (n = 9) in a preliminary study [45]. Findings suggested that while analysts felt that maps are a useful tool, their existing mapping software, level of exposure, or skill set with regard to this type of software do not adequately meet their data synthesis needs. Moreover, some analysts indicated that promoting the subsequent use of maps for decision-making by managers can be difficult because some managers do not know how to interpret this data. The findings also revealed that the OEYCs are an ideal setting to study mapping/maps as innovative research transfer tools because: 1) the barriers identified by data analysts are not insurmountable to overcome with a refined mapping tool and training; and 2) OEYC data analysts and managers are in a setting where mapping/maps are encouraged by the related Ministry. Hence, the overall context represents an ideal setting for map use.

Recognizing the opportunities and barriers that exist to further integrate mapping into the decision-making process, this project was developed in two distinct phases. Phase 1 involves the iterative and collaborative design and implementation of the web-based mapping software (EYEMAP) based on a participatory design (PD) process through a modified user and task analysis [46] and cooperative prototyping [47,48].

A case study design [49] is being used whereby the case (i.e., the unit of analysis) is defined as the data analyst and manager dyad in selected OEYCs. Multiple cases are used to support the reliability of findings. Presently, nine producer/user pairs are participating in this project. The goal through these two phases is to minimize the barriers to map production and use by data analysts and their managers. In other words, when the evaluation is conducted at the end of the project, the aim is to ensure that reasons for *not *using mapping or maps are not related to challenges in using the web-based mapping software (by data analysis) or in understanding how to properly read and interpret spatial data (by managers).

The conceptual framework guiding this study – the Ottawa Model of Research Use (OMRU) [50,51] – links the development of the two project phases. The OMRU assembles diverse aspects of the process of health services research use into a simple but widely applicable framework for assessing barriers to utilization. In the OMRU, the utilization of research is dependent on three sources: the innovation, the potential adopters, and the environment [50]. Perceptions of the attributes or characteristics of the innovation can influence potential adopters' decisions to use the innovation in either positive or negative ways. Potential adopters (the producer and user of maps) have particular motivations, skills, and attitudes that may affect uptake. The environment also contains structural and social influences that may foster or impede the uptake of an innovation (mapping for data analysts; maps for managers). The OMRU framework's key propositions suggest two main courses of action for this project. First, the barriers and facilitators of the innovation associated with the environment, the potential adopters, and their perceptions about the innovation need to be identified and overcome for optimal uptake and use. Second, the research transfer strategies (i.e., the interventions) to promote use of the innovation ought to be tailored to address the barriers and emphasize the potential supports associated with the environment, potential adopters and the innovation [50]. The model also directs attention to the need to monitor the implementation of interventions and uptake of the innovation over a period of time due to the dynamic nature of research use.

## Results

Participatory design involves gathering information on each user and/or task, including experience level, capability, data access, data requirements and steps involved related to the task. As research transfer occurs within a social system [52], it is necessary to identify the individual and organizational facilitators and barriers of creating and using local data. The user and task analyses conducted in this phase helped us to refine a collaborative mapping prototype and associated support system to meet the specific needs of data analysts and managers. These analyses also provided important information on research transfer issues between producer/user dyads in these OEYCs.

With the data analysts, the project team was interested in the technical aspects of their data and mapping perceptions and needs, and what functionality they would like to see in mapping software. For the managers, the user and task analysis focused on evaluating skills in map reading and spatial data analysis, determining the type of maps they would want to receive for decision-making purposes, as well as the assessing the perceived usefulness of maps to represent local data.

The first meeting was important in establishing trust. Data analysts were initially skeptical about participating. Their concerns related to the uncertainties associated with a research project that had not yet received sufficient research funding to carry out the work, as well as putting time and energy into learning a web-based tool that might not be available for their use after the project ended. Despite these concerns, relationship building during this first meeting engendered initial buy-in: data analysts left feeling sufficiently confident and interested in the project to agree to assist in the design process, with the full knowledge that it may never be completed. At this first meeting, based on a group discussion with participants, the project team collected participants' 'wish list' of what the ideal GIS tool would be for them. Participants also expressed what they felt were the limitations of the mapping software that they used (for those who had access to such software), as well as what basics they needed a mapping tool to do in order to assist them with their day-to-day tasks.

The meeting revealed wide variation in the extent to which data analysts used mapping software. Moreover, there was no consistency or standardization among OEYCs in the software being used. Consequently, very little sharing of maps and data occurred among data analysts. The limitations of existing software related to software inaccuracies (e.g. placing land data points in rivers), the complexities and expense involved in using more sophisticated GIS software like ArcGIS/ArcView or MapInfo, and the inability to print-preview resulting in many map iterations to have the printed form match what analysts saw on their screens. Another major challenge for analysts was acquiring a precise and relevant local geolocating database (i.e. turning address information into latitude and longitudinal coordinates that can be mapped). In particular, the postal code areas were often too large and not specific enough to be relevant in rural locations, while complete street information plagued researchers in rural communities consistently and urban communities intermittently. For example, one participant stated, "A lot of times what happens in rural areas, you'll see a small town and you'll actually see the outline of streets and stuff. They don't even have names on them or anything. So you will see a point that is maybe on the wrong side of town or something."

Those analysts that produced maps but who did not have access to any GIS software reported that they were forced to rely on a GIS department within their organization. While such a relationship facilitated the production of maps for data analysis purposes, the participants still faced a number of challenges. Specifically, participants commented on competing priorities being the largest barrier – the data analyst needs compared to the larger organizational demands placed on the GIS department – that often resulted in delays (upwards of days to weeks) between putting in a request and receiving a map. For example, in the words of one data analyst participant: "by the time we would get the map from the GIS department, it no longer met our needs because our managers' needs changed where they wanted different data mapped." One manager participant explained, "To try to access any mapping is a real challenge and it's a cost issue on top of that, a time issue. So something like this where a data analyst can do it for the OEYCs, that's going to be really useful."

Following this initial meeting, it was determined that the primary and guiding requirements for any tool developed for these participants must: 1) be easy to use and have a low learning threshold; 2) have some mechanism to protect proprietary and sensitive health data if it is web-based; 3) permit limited and controlled sharing between participants such that not only was sharing possible, but that the receiving participant would not need the original software in which a map (for example) was created; and, 4) ensure that those with minimal proper training in creating maps could easily avoid breaking cartographic conventions. By way of illustration, the system should not allow users to represent count data, such as number of children under six, as a choropleth map (i.e. a thematic map that best depicts data category classes through shading), when visualizing such data as proportional symbols is more appropriate. The framework for the mapping tool, user interface, and functionality was designed following the above requirements. Moreover, after reviewing commercial and open-source software options it was clear that open-source web mapping would be the most beneficial from a cost perspective – given the needs of the participants and budgetary limitations of the pilot project.

EYEMAP was developed using the University of Minnesota's MapServer [53-57]) and map tool resources. MapServer is fully Open Geospatial Consortium (OCG) compliant and as such, easily interacts with other standard web mapping services and software. In particular, resources at MapTools.org, hosted by DM Solutions Group [58] were heavily leveraged in the development cycle. MapServer was used in a Linux environment in combination with Perl and C++, to build the prototype mapping of EYEMAP for the OEYC data analysts.

The second and third meetings involved a similar process. The second half-day meeting with participants was to conduct some proof of concept demonstrations of open-source software and what was being developed for participants. This second meeting was important as it allowed us to refine the participants' 'wish-list' into something that could be functionally implemented. The list of mapping functionality and content that was identified included:

1. A common base-map including political units, places, roads, and major water bodies for the entire southern Ontario region.

2. The ability to geocode address-based client data.

3. The ability to show point (a.k.a. push-pin) maps of clients and services.

4. The ability to produce choropleth maps of rate data.

5. The ability to produce graduated symbol maps of count data.

6. The ability to produce unique value maps for nominal data.

Ensuring a secure platform was considered an essential constraint to the OEYCs' use of the web mapping tool and was the primary focus of email discussions with participants before the third meeting. Using web-based software requires a secure interface for data analysts to upload their local-level proprietary data. This issue arises due to confidentiality of health related datasets, but more importantly in this project due to different data-sharing agreements between the OEYCs in southern Ontario. As such, data analysts with sharing agreements wanted to utilize the web-mapping software to share datasets that cross jurisdictional boundaries but at the same time needed to ensure that only those data analysts with agreements in place could share the data. The users were instructed (reinforced with warnings from the software) to only share with people with whom they have signed agreements. The software enforces a security model where information (data or maps) on the system is only available to a user if the owner (another user) of the data has explicitly shared it. Once shared information is available to the non-owner, the non-owner has access to this file. The non-owner operates under an honour system to not share the file with another person without the owner's express permission. All participants are aware of the risks involved (i.e., improper sharing) regardless of the owner's original desire. This security model reinforces what naturally occurs when information is published to a private audience.

The third meeting presented a preliminary EYEMAP prototype to obtain additional feedback from participants. Obtaining more specific participant feedback at this stage was important to ensure that any design constraints and assumptions were made clear to the data analysts. For example, different GIS software in use by various data analysts (MapInfo, ArcGIS etc.) utilize different spatial data formats for the same spatial objects (e.g., a point set showing medical centers) because of vendor data formats. For example, ESRI shapefiles can contain a single feature layer, like roads, but to interpret and display the file requires the software have access to three files with the same name but different file extensions. Browser security limitations in accessing local file systems constrained the design and functionality of the upload interface in EYEMAP. By way of illustration, for a data analyst to share a shapefile with a colleague requires that s/he explicitly point the EYEMAP upload interface to the three necessary files manually. This form of data upload is mandatory in secure web software but was foreign to a native ESRI user whose software is designed to view all three files as one. This third meeting was fundamental to explain some of the oddities such as the upload interface to the data analysts.

To complete the PD process it was important to bring in both data analysts and their managers. This final meeting consisted of two parts. First, the project team met with data analysts and managers separately. Data analysts held discussions with the two primary developers from the research team to assess more technical elements such as those described above. For the managers, from whom the project team still required buy-in for their full participation in the project, it was important to collect some baseline data. With the managers, the project team needed to assess their comfort level with maps (analyzing spatial data), and the type of data they would like to have mapped to support decisions. As one manager stated:

We haven't used maps just because we haven't had the ability to before the onset of Best Start. She has developed charts and whatnot to convey the information...So as far as being able to have a closer look and a more effective look at what is happening with our community, and if there are friends in our various neighborhoods where we either need more services, less services, we need to look at providing services in a more effective way, or reallocate resources. Our hope is that at the end of the day, this work [mapping project] will assist us in doing work more efficiently and more effectively.

The project team also assessed whether or not they perceived the spatial representation of local data in the form of maps as useful (i.e. compared to data charts in graphic or tabular form). One manager stated "I mean graphs are good but mapping is evidence. Hard fact evidence. And I think that is how we like to see it."

Second, using mock-data (since proprietary OEYC data could not be accessed), participants viewed a presentation of the types of decisions that could be supported by local level data. This presentation served two primary needs: to both demonstrate how maps can be a decision-support tool and to provide data analysts and managers with a proof-of-concept of the collaboratively designed web-based mapping software. Discussions during this presentation also served to ensure that managers were willing to permit a more honour-system based network of sharing local data; which they were as per their explicit signatures on University of Ottawa Research Ethics forms that outlined the benefits and risks of such a sharing system.

## Discussion

Phase 1 of the project is complete. Overall, this phase has served to assess the perceived barriers to mapping and maps related to the innovation, potential adopters and environment [50], as well as to elicit attitudes about the value of producing maps and the value of maps for research transfer. The majority of EYEMAP's functionality has been operationalized and participants have received two one-day training sessions on its use. Key components of the training sessions included hands-on exercises and the distribution of a desk-manual to participants as a reference document. EYEMAP is also equipped with an online 'Help' support that is targeted to aid less spatially literate users in making correct choices while providing answers to typical use problems. Recognizing that the collaborative design process is an ongoing relationship, EYEMAP is being incrementally developed based on the early and initial use by participants in their real-work setting. This has mainly involved adding server side functionality and incorporating this functionality into the working EYEMAP interface. Any future developments to EYEMAP will be done within the existing interface and will preserve existing functionality.

The successes and difficulties in Phase I with respect to the collaborative interface and software development of this project are worth noting. Both the PD process and two key features of EYEMAP – data/map sharing functionalities and the interoperability of the tool – are considered as key successes in this project. Some challenges represent potential limitations associated with the PD process among the developers and the users. These will each be discussed in turn.

The success of developing a software tool by inviting suggestions and input through a PD process cannot be understated. The users' motivation for continued successive iterations of this development was only possible through proof of concept and prototype demonstrations. The success in using this approach has been remarkable given the substantial challenges facing this type of software development. From a technical standpoint, the open-source tools used by the development team (MapServer and Chameleon) were both a facilitator and a barrier. Two key components of the PD process was the users' request for map/data sharing capabilities across jurisdictions, particularly where there was overlap between the delivery of services among some OEYCs. Permitting map/data sharing required that these be delivered on a secure platform and that there be a degree of interoperability where various map formats could be viewed through EYEMAP without requiring the ownership of proprietary GIS software. As a model that reinforces what naturally occurs when information is published to a private audience, the web-based data sharing component is innovative. The project team foresees the data-sharing model and the interoperability as being the major functions for which EYEMAP will be used by analysts as is evidenced by the creation of new data sharing agreements between different OEYCs. In this way, the development of EYEMAP bridged a barrier to free exchange across jurisdictional boundaries, and serves as an example of the fruitfulness of participatory GIS process.

Nonetheless, these advances have had some associated challenges; some that affect the developer, some that affect the users, and some that affect both parties due to the nature of the PD process. On the developer side, challenges have included the length of programming time needed to make MapServer operational in a secure data sharing paradigm. At a much larger scale, challenges were experienced in obtaining personnel skilled in web application development and GIS; personnel skilled either in web application or in GIS have been found, but not both. Training in both of these technical fields should be encouraged for researchers and trainees (i.e., graduate students) conducting work in this area. Certainly, to avoid these problems and the time and energy required to develop a new application, it is recommended to, whenever possible, work within the constraints of an existing software package and to adapt the interface to the users' needs. This option, however, was not possible in this study context. On the user side, one challenge (that remains to be overcome) is the need for framework or base-data sharing among the OEYCs in the study. While the OEYCs can create spatial/aspatial data sharing agreements between themselves for data they collect, these agreements do not necessarily apply to third party data sharing agreements or licenses that individual OEYCs may have with providers of value-added data (e.g. socio-economic datasets from Statistics Canada). This is a hurdle that no technological tool can address. A significant challenge affecting both developers and users was associated with the implementation of the PD process. Despite the project team's familiarity with the functions, roles, and purposes of the OEYCs and their managers, a stronger focus on spatial literacy and more research into the decision-making process at the OEYC managerial and operational level at the outset would have streamlined the design process. The developers required more explicit information regarding the functionality users needed, however to get this, users required a better understanding of the types of questions a GIS can help answer (i.e. spatial vs. aspatial). This issue became apparent during the PD process and user testing, and often led to changes of the prototype on an ad-hoc basis.

A broader limitation within the PD process is also one of its greatest strengths: the interaction between developers and users in real time. Participants are working in a provincial context that has undergone tremendous programmatic changes at the governmental level. This, combined with other organizational realities, namely staff turnover and changes in leadership, has meant that not all of the original participants involved at the beginning of the project have remained throughout the PD process. In fact, while the data sharing functionality one of EYEMAP's is considered as one of its hallmark features, and one that is appreciated among users involved from the beginning, some of the newer users, particularly those that reflect some of the newer programmatic changes within the Ministry of Children and Youth Services (the Best Start Program), are less keen. For these newer users, greater security than was made possible through the development of EYEMAP is desired. In its absence, for these users, there may be an initial preference to eliminate the sharing functionality altogether.

Despite these challenges, greater uptake of this web-based mapping tool is expected among project participants and possibly a future provincial roll-out as a result of the participatory design process provided that EYEMAP can meet the evolving needs of participants. PD approaches, by their very nature, encourage buy-in to the development process, better addresses user-needs, and creates a sense of user-investment and ownership in both the EYEMAP product and the research project. These will be evaluated more concretely at the end of Phase 2 of the project; however, there is no way to properly test the effectiveness of the PD process.

Moreover, evaluating the methods and *process *of an implementation project like Phase 1 of EYEMAP is just as valuable as assessing future *outcomes *from the uptake of a tool like EYEMAP to support the use of evidence in decision making, which will be undertaken through Phase 2 of the research project. In this way, unfavourable outcomes can be properly attributed to either poorly implemented innovations or to participants' lack of innovation uptake. Phase 2 includes the intervention and implementation phase of the innovations. In the initial project proposal, it was felt that to facilitate use of the innovations (mapping software for data analysts and maps for managers), only two support interventions in selected OEYCs would need to be implemented for a period of twelve months.

The purpose of these interventions are to encourage use of mapping software and maps, as the primary project focus is to study the use of these innovations. For the data analyst, the intervention was to only include a support system composed of a training manual, an on-line support system and in-person training sessions to use the refined mapping software. However, given the variability that exists among the computer skills of different data analysts it is important that further one-on-one training is provided as the analyst works through some specific tasks in their organizational setting. This will enable future implementation decisions for how future training sessions may need to be developed for a larger provincial roll out of this program. For the manager, the intervention remains unchanged and will comprise a series of one-on-one informal training sessions about interpreting maps. The first two sessions will introduce basic mapping concepts and methods, how maps can be used and misused in representing data, and the types of questions that maps can help one answer. These ideas and concepts will be introduced using the EYEMAP software and examples will come, whenever possible, from potential OEYC scenarios. Basic training in EYEMAP will also be provided here. The third and forth sessions will be more focused, working through a series of relevant scenarios where mapping could help answer a question or address a problem specific to a given OEYC activity. An example might include where best to locate a new service given specific population characteristics, gaps in services, and public transportation access. The fifth and final session will focus on various practical issues including various data sources (the national census, government health surveys, etc.), data quality and access, geo-referencing, assumptions of causality, and pitfalls of spatial association (e.g. ecological fallacy and modifiable aerial unit problem).

Innovation uptake and impacts will be monitored and evaluated following the interventions through focus groups, field notes and an activity logger that will monitor use. In addition to these one-on-one training sessions with managers, they will receive audit reports of EYEMAP use vis-à-vis the use of participating OEYCs. These will be used to serve as a mechanism to inquire about the relative use of a particular OEYC to ensure that there are no technical problems that needs to be addressed on the server side. Focus groups will also explore the attitudes of data analysts and managers toward mapping and maps, particularly in terms of how comfortable they are with maps compared to their current use of tables and other data presentation methods.

## Conclusion

This paper presents the developmental phase of an interdisciplinary, collaborative GIS project. Its focus has intentionally been on the methods needed to help promote use, acceptance and ownership of a GIS tool to support child health decision making by involving users throughout the development phase. The iterative consultation of users, through PD processes, is a strategy encouraged by both computer sciences and research transfer. The purpose of collaboration, theoretically, is to develop a tool that is more aligned with the users' needs. From a research perspective, the collaboration has made us, the research team, more sensitive of the users' working environment. Further, the groundwork has been laid for future joint projects around the use of maps to support evidence-based program planning in the child health sector.

## Competing interests

The author(s) declare that they have no competing interests.

## Authors' contributions

All six authors actively participated in the development of the ideas and writing of the article. All authors actively participated in the conception of the research design. SMD and AK were the leads on obtaining research funds for this project. JM developed the EYEMAP web-based tool, developed the training manual and trained participants in the use of the tool. SMD, AK, MS and EJC wrote different sections of the manuscript while others, namely SMD, AK, EJC and IDG actively edited the manuscript to ensure a common voice throughout. EJC and IDG played key roles in editing for logical flow. All authors have given approval for the final version of the version to be published.
